# Notes and News

**Published:** 1901-03-30

**Authors:** 


### NOTES AND NEWS.

THE King has consented to become patron of the Home Hospitals Association.

THE Queen has consented to continue patron of the British Home for Incurables.

THE King has graciously consented to continue to be patron of the Metropolitan Hospital, Kingsland Road. Mr. A. H. Allhusen, M.P., will preside at the festival dinner in aid of the funds of the hospital, to be held at the Whitehall Rooms on April 22nd next.

THE legislature of New York State are making stringent regulations in regard to the sale of poison. All bottles in which specified poisons are sold are to be marked "Poison," and the address of the seller clearly printed. The sellers are to keep a careful record of all poisons sold, together with the date and the name and address of purchaser.

THE Town Council of Scarborough are about to erect an isolation hospital. Tenders for the building which have been received will, after some modifications reducing the cost, amount to an expenditure of £500 per bed. Naturally some of the members of the Council think this a high figure, but it does not appear that the plans have been referred to an expert, and it is probable that Scarborough will submit to the enormous expenditure asked of it.

THE Italian Congress of Internal Medicine will be held at Pisa in October.

A NEW ward for twenty beds has been opened at the Stanley Hospital, Liverpool.

SIR WILLIAM BROADBENT has been created a Knight Commander of the Victorian Order, and Dr. A. R. Manby is to be a member of the Fourth Class of the same Order.

SYDNEY, New South Wales, is again the unfortunate victim of an epidemic. This time it is suffering from smallpox, which is a disease that Australia has taken great pains to keep at bay.

LIVERPOOL and Manchester are stirring their Parliamentary representatives to move for the exemption of charities from rates. Thus the hospitals will benefit by the agitation which will include them.

THE Lord Mayor of Liverpool has received already half the sum of £20,246 required to free the local hospitals from debt in 1899, which he appeals for as a method of marking the commencement of the new century.

ST. MARY'S HOSPITAL has just received £25,000 from the executors of the late S. R. Zunz, Esq., on condition that the authorities raise within six months the sum of £50,000 to enable them to complete forthwith the Clarence Memorial Wing.

AN attempt is being made by the City Councils of towns with seaports to secure from the Government the expenses incurred by the Port Sanitary Authorities, it being maintained that the benefits are to the general community, not solely for the local inhabitants.

IT is serious news that the plague has attacked the troops at Capetown. So far we have reports that one soldier of the Queen's Regiment at Simonstown, one in the 1st Royal Irish Regiment at Greenpoint Camp, and one in the Royal Garrison Artillery at the Castle with other Europeans have been attacked in addition to the native population which has suffered.

THE Newcastle Infirmary seems destined to be the recipient of munificent sums of money. The committee have recently received a gift of £100,000 from Mr. and Mrs. Watson-Armstrong in memory of the late Lord Armstrong. The gift is without conditions, it being the sole wish of the donors that the work of completing the institution, which was founded to commemorate the late Sovereign's glorious reign, should proceed without delay. The conditions attaching to the Hall bequest are still under dispute.

IT has been decided to establish a sanatorium for consumptives at Winsley, near Bath, to serve the counties of Somerset, Gloucester, and Wilts. The building will be situated on a high hill, overlooking the Avon. It will face south, and possess grounds of 53 acres. The sanatorium is intended for the class intermediate between that which can pay three guineas a week and upwards, and the rate-aided poor. The scheme should prove so useful that we hope that it will be found possible to carry it to a financial success. Undoubtedly such institutions are much needed.

